# Affirmed gender shapes virtual embodiment: a phenomenological account of ownership and agency across gender identities

**DOI:** 10.1093/nc/niag047

**Published:** 2026-08-14

**Authors:** Marina Leggieri, Alessio Borriero, Tommaso Ciorli, Maria Pyasik, Selene Schintu, Sarah Finzi, Maria Teresa Molo, Lorenzo Pia

**Affiliations:** Department of Psychology, University of Turin, Via Verdi 10, 10123 Turin (IT), Italy; Department of Psychology, University of Turin, Via Verdi 10, 10123 Turin (IT), Italy; Department of Psychology, University of Turin, Via Verdi 10, 10123 Turin (IT), Italy; Scientific Institute, IRCCS E. Medea, Via Cialdini, 29, 33037 Pasian di Prato UD, Udine, Italy; CIMeC - Centro Interdipartimentale Mente/Cervello, University of Trento, Corso Bettini, 31, Rovereto, 38068 TN, Italy; Department of Psychology, University of Turin, Via Verdi 10, 10123 Turin (IT), Italy; Centro di Psicoterapia Cognitiva e Servizi alla Persona C.so Galileo Ferraris 110 - Torino, Torino, Italy; Fondazione Carlo Molo Onlus, Via della Rocca 24 bis, 10123 Torino, Italy; Department of Psychology, University of Turin, Via Verdi 10, 10123 Turin (IT), Italy

**Keywords:** embodiment, gender identity, bodily self-consciousness, multisensory integration, virtual reality

## Abstract

Gender identity plays a crucial role in how we come to feel our bodies as our own. When the inner sense of gender does not align with bodily appearance, the resulting tension can shape both conscious and pre-reflective aspects of embodiment. In this study, we examined how gendered self-representations influence bodily self-consciousness using the Full-Body Illusion in immersive virtual reality. Eleven transgender and eleven cisgender participants embodied both gender-congruent and gender-incongruent avatars viewed from a first-person perspective under visuomotor synchrony. Participants rated their sense of ownership and agency using the Avatar Embodiment Questionnaire, and completed the Utrecht Gender Dysphoria Scale–Gender Spectrum to assess dysphoria and affirmation. All participants experienced a convincing illusion of embodiment. Yet, only transgender participants showed a clear difference between conditions, with stronger embodiment for gender-congruent avatars. Bootstrapped analyses confirmed higher ownership and mirror-ownership scores in the congruent condition, and correlations revealed that dysphoria was positively associated with mirror-based embodiment, whereas affirmation was negatively associated with agency. These findings suggest that the sense of owning and controlling one's body may be influenced not only by multisensory integration but also by its alignment with one’s affirmed gender. Gender identity thus may emerge as a deeply embodied and dynamically instantiated dimension of minimal selfhood, bridging the sensorimotor and the phenomenological layers of bodily consciousness.

**Significance Statement**

Bodily self-consciousness is generally understood to arise from multisensory integration, yet the contribution of gender identity to this process has remained largely unexplored. Using immersive virtual reality and the Full-Body Illusion, we provide the first experimental evidence that the congruence between a perceived body and one’s lived gender identity selectively modulates embodiment in transgender individuals. These findings suggest that gender identity-related representations interact with the sensorimotor mechanisms supporting conscious bodily experience, extending current models of minimal selfhood beyond multisensory integration alone. By integrating research on embodiment, gender identity, and virtual reality, this work establishes a novel experimental framework for investigating the embodied basis of identity and provides a foundation for future studies on the cognitive and neural mechanisms linking gender identity, embodiment, and consciousness.

## Introduction

Gender identity, namely, the set of personal beliefs and self-representations about one’s own gender, is derived from the socio-cultural meanings of masculinity and femininity and contributes as a category to how we perceive our own body. Across the lifespan, “we can choose consciously from among the many cultural features of gender to embed new bodily habits into our sensorimotor (neuromuscular) system. Even without conscious choice, however, many cultural features of gender shape how our bodies function” (Fausto-Sterling 2019, p. 5). In line with the embodied cognition framework, an unconscious and interior sense of gender/sex gradually settles in the body as a “biosocial sediment” (Fausto-Sterling 2019, p. 6). The term “gender/sex” was first introduced by van Anders and Dunn (2009) to address the inherent inextricability of the categories of gender and sex, and recognize their “entangled co-constitution” (DuBois *et al*. 2025, p. 5). Early-life dyadic interactions convey the first presymbolic representations of gender through sensorimotor experiences, which vary in tone depending on the caregiver’s gender (i.e. maternal vs. paternal interactive style). The autonomic, neuronal, and hormonal systems of each partner in the dyad are affected by these exchanges, and, over time, they substantiate the child’s symbolic representations of gender, capitalizing on sensorimotor information as a source of meaning while interacting with other human bodies (Fausto-Sterling 2012). The cultural and normative structures of heteronormative gender/sex are carefully and implicitly imposed on individualized nervous systems and bodies, which are then interpreted through the lens of these categories. In the meantime, developing our self-consciousness within an individualized body dialectically interacts with such cultural norms. A narrative of our gendered self “emerges from three inextricable modes of bodily activity: self-regulation, sensorimotor coupling and intersubjective interaction” (Thompson, 2005 in Fausto-Sterling 2019, p. 8). These developmental processes shape gender as a dynamic, deeply embodied, and intersubjective dimension of identity.

The influence gender identity exerts on the bodily self is stressed by gender dysphoria and more specifically body dysphoria, namely, the distress resulting from the mismatch perceived between one’s gender identity and sexually dimorphic bodily features. This mismatch may generate profound discomfort and self-directed negativity, leading some individuals to disengage from their physical body and bodily signals, resulting in a total break between their sense of self and physical body (Cooper *et al*. 2020)—a phenomenon commonly referred to as disembodiment. Disembodiment is characterized by a low sense of ownership (i.e. “I am the subject of a certain experience/movement”), and a low sense of agency (i.e. “I am the executor of a certain action, and it is the result of my will”) (Gallagher 2000, Tsakiris *et al*. 2007). Since our experience is grounded in a sensorimotor apparatus, the sense of body ownership and of agency deeply rely on multisensory integration processes (Gallagher 2023). Integration of multisensory signals that are precisely localized in time and space, originating both within and outside the body, and perceived from a first-person perspective (1PP), gives rise to the minimal self or minimal phenomenal selfhood (Gallagher 2000, Blanke and Metzinger 2009, Zahavi 2017). Therefore, misregulation in the coordination of multisensory signals can profoundly affect the sense of selfhood, possibly triggering states of disembodiment (Ciaunica *et al*. 2022).

These dynamics are particularly relevant when considering embodiment in transgender and gender-diverse (TGD) individuals, as they are often associated with dysphoria or discomfort in relation to one’s body. Such experiences are largely socially and stigma-induced, rather than intrinsic to gender diversity itself (Richards *et al*. 2016, Coleman *et al*. 2022), since normative structures generate feelings of unbelonging by promoting sexual dimorphism as a scientific truth rather than a regulatory discourse on bodies (Butler 1993, Walsh and Einstein 2020). Stigma toward LGBT people creates ‘minority stress’, on a distal–proximal continuum that impacts every aspect of life, from everyday microaggressions to interiorized transphobia (Meyer 2015).

Within this context, phenomenology as a qualitative research methodology is particularly well suited to unravel how societal expectations and our bodily self-consciousness interact to shape our sense of self, offering to delve into the subjective phenomena occurring within one’s own consciousness (Ekici *et al*. 2020) when embodying a virtual avatar. Here, we wanted to capitalize on the experience of perceiving from a 1PP a gender congruent or incongruent virtual body while keeping a quantitative measurement of such experience through the Avatar Embodiment Questionnaire (AEQ; Pyasik *et al*. 2023).

The Full Body Illusion (FBI), a body ownership illusion that focuses on a first-person experience of the bodily self, investigates subjective feelings of ownership and agency towards a different body while immersed in a highly controlled environment that allows causal manipulation. The FBI relies on integration of multisensory signals, in which spatially and temporally concurrent multimodal sensorimotor information is integrated to form unified percepts that stand at the basis of our bodily self-consciousness (Lenggenhager *et al*. 2007, Petkova and Ehrsson 2008, Slater *et al*. 2010, Maselli and Slater 2013). Through synchronous bimodal stimulation, multisensory integration can produce the illusion that the artificial body and the real one coincide. Following recent developments in virtual reality application in cognitive neuroscience research (Gonzalez-Franco and Lanier 2017), the FBI paradigm exploits a head-mounted display (HMD) to—quite literally—put participants in the shoes of a virtual avatar, with a 1PP and bimodal synchronicity (i.e. visuomotor or visuotactile) (Maselli and Slater 2013). The potential of such manipulation has been explored extensively with various bodily alterations (Banakou *et al*. 2013, Peck *et al*. 2013, Tacikowski *et al*. 2020, Pyasik *et al*. 2023, Dimakopoulou *et al*. 2026), showing how body ownership affects a wide range of cognitive processes (for an extensive review on the topic, see Pyasik *et al*. 2022), yet no study so far has addressed the effects of a gender-congruent virtual embodiment on transgender and gender diverse people or how it interacts with gender dysphoria. This study aims to investigate the effect that embodying a virtual avatar characterized by a stereotypical masculine or feminine gender expression has on the bodily self and own-body perception. Considering virtual embodiment as grounded in multisensory integration processes, the FBI may provide a framework for investigating how gender identity and multisensory integration interact to form bodily self-consciousness. To this end, we administered a gender-congruent and a gender-incongruent FBI to cisgender and transgender individuals to investigate how gender-focused alterations in the bodily self influence body ownership and agency, and how these effects would correlate with different levels of dysphoria and affirmation. We expected the embodiment illusion to be successful in 1PP with visuomotor synchrony in all participants, since embodiment procedures have proved to be successful even with mismatching ethnicity, age, or gender (Banakou *et al*. 2013, Peck *et al*. 2013, Tacikowski *et al*. 2020, Pyasik *et al*. 2023). However, in transgender individuals, we hypothesized gender incongruency to significantly attenuate feelings of ownership and agency over the virtual body.

## Materials and methods

### Participants

Eleven transgender individuals (*Trans*; 23.7 ± 3 years, range 20–29) and 11 cisgender individuals (*Cis*; 23.5 ± 3.6 years, range 19–29) with no history of neurological or psychiatric disease provided written informed consent to participate in the study. Transgender participants were recruited with support from the Interdepartmental Center for Gender Identity Disorders at Molinette University Hospital (Turin, Italy). Information about participants’ gender identity was gathered via a self-report questionnaire on various demographic information, and only individuals who identified as binary transgender or cisgender were included in the sample. Participants in the transgender group included both people who had received gender affirming hormone therapy and people who had not. Gender dysphoria was assessed in all participants with the UGDS-GS (McGuire *et al*. 2020). The UGDS-GS assesses levels of dysphoria with 18 items subdivided into two subscales, Affirmation and Dysphoria, on a 5-point Likert scale. The study was approved by the Bioethical Committee of the University of Turin (protocol number 0345740).

### Procedure

**Figure 1:**
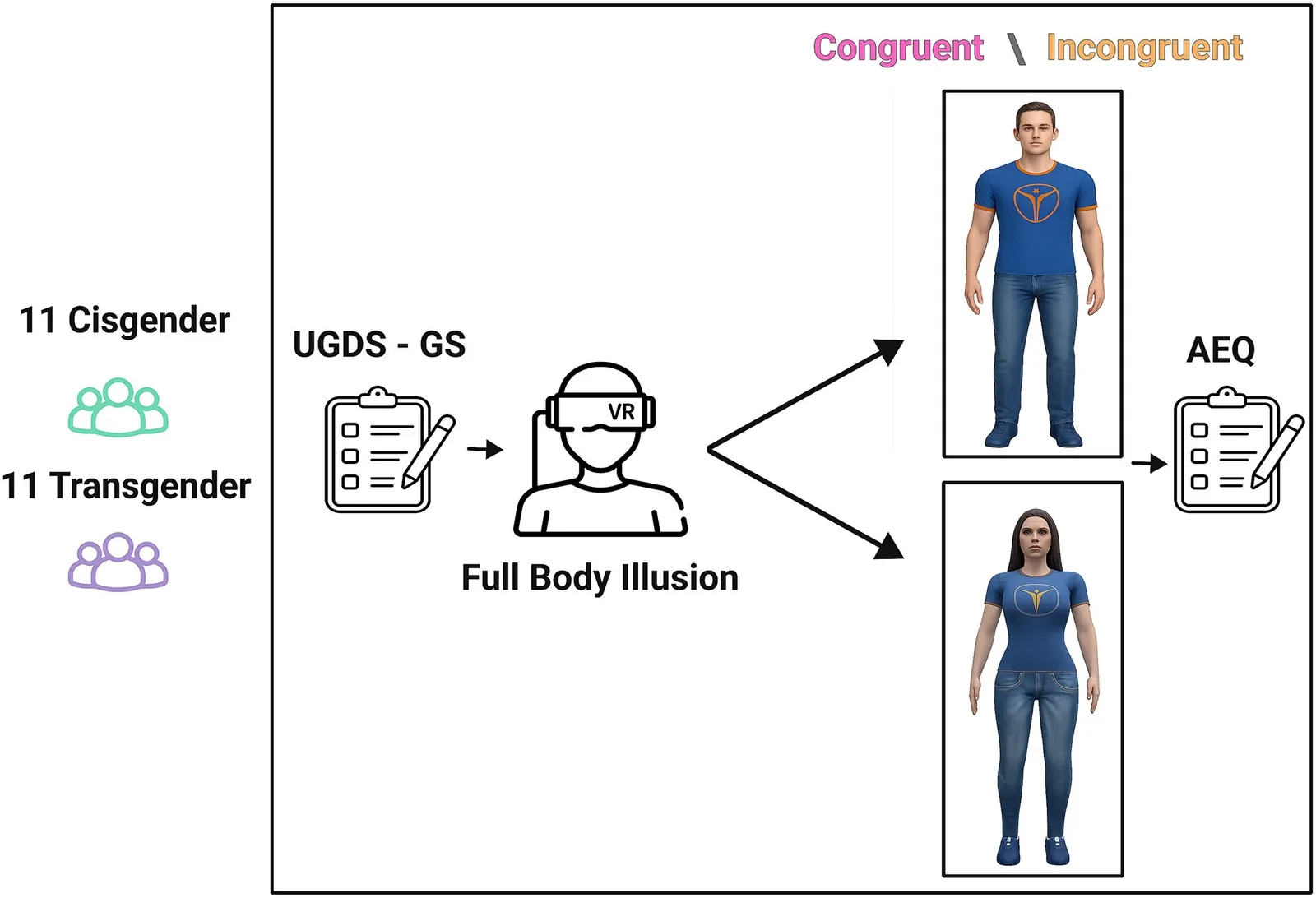
Experimental design overview and procedures. All participants (11 Cis; 11 Trans) completed the UGDS-GS questionnaire before participating in two FBI sessions: one with a gender-congruent avatar and the other with a gender-incongruent avatar. The Avatar Embodiment Questionnaire was administered after each session.

As shown in Fig. 1, all participants completed two embodiment sessions, scheduled at least 1 week apart to minimize potential carryover effects from the preceding session (Banakou *et al*. 2016). In one session, they experienced the gender-congruent FBI, while in the other, they underwent the gender-incongruent condition. The order of conditions was counterbalanced across participants. While still being immersed in the virtual environment, the experimenter verbally administered the AEQ items in a randomized sequence. Prior to both embodiment sessions, participants also provided demographic information and completed the UGDS-GS assessing gender dysphoria and gender affirmation.

### Virtual scenario and embodiment procedure

The virtual environment was developed in 3D Studio Max 2023 (Autodesk Inc.) and implemented in Unity 2021 (http://unity.com). It consisted of a virtual office room furnished with basic items and a large mirror. Avatars were generated using MakeHuman (http://www.makehumancommunity.org/) and included both male-presenting and female-presenting models, with secondary sexual characteristics maximized. Participants experienced the scenario through a Meta Quest 2 HMD (www.meta.com; resolution: 1832 × 1920) and handheld motion-tracking controllers, which enabled visuomotor synchrony.

Each embodiment session lasted ~10 min. Upon entering the virtual environment, participants immediately viewed their virtual body reflected in the mirror in front of them, from a 1PP. They received instructions on the range of performable movements: while their feet were to remain still (as their motion was not tracked), they could move their head, torso, arms, and hands, and bend their knees freely. Then, participants were given 2 min to autonomously explore the environment and familiarize themselves with their virtual body. Afterward, the experimenter guided participants through a standardized sequence of movements intended to focus their attention on the avatar (i.e. body inspecting from a 1PP, spreading their arms and moving them in different directions while observing themselves perform these movements in the mirror, etc.). The session concluded with two additional minutes of autonomous exploration of the virtual environment and of self-body inspection, followed by the verbal administration of the AEQ. Particular attention was devoted to both direct first-person inspection of the virtual body and observation of the avatar through the virtual mirror, as these perspectives contribute to distinct aspects of embodiment. Accordingly, the AEQ included separate measures of ownership experienced from a 1PP, ownership of the avatar as seen in the mirror, and agency over the avatar’s actions.

### Avatar Embodiment Questionnaire

The AEQ, administered at the end of the FBI, consists of 12 statements assessing the quality and the magnitude of the embodiment illusion on six dimensions: location, ownership, ownership of the avatar as seen from a third-person perspective in the mirror, appearance, sense of agency, and sense of will over the avatar’s movements. In the present study, mirror ownership was considered not merely as a perceptual feature of the virtual environment but as a distinct embodiment dimension reflecting the experience of recognizing and attributing the avatar’s reflected body to oneself from an external, third-person perspective. A corresponding control statement mirrors each real statement, and participants rate each on a 7-point Likert scale ranging from −3 (complete disagreement) to +3 (complete agreement), with 0 as the scale’s midpoint, standing as “I do not know” (Pyasik *et al*. 2023).

### Data analysis

For the analysis of embodiment quality and quantity, we focused on a subset of three AEQ dimensions: Ownership, Mirror Ownership, and Agency. These dimensions were selected on an a priori theoretical basis, as the main hypotheses of the study concerned the modulatory effect of virtual gender congruency on body ownership and agency. Alongside pairwise comparisons of these single dimensions between groups and conditions, we applied a principal component analysis (PCA) to the 3D space composed by the Ownership, Mirror Ownership, and Agency dimensions of the AEQ, identifying a principal dimension that accounted for the largest variance in embodiment assessment (Abdi & Williams, 2010, Solanas *et al*. 2011). In this way, the first PCA component provides a concise scalar representation of the magnitude of embodiment. The adequacy of this representation was evaluated based on the proportion of variance explained by the first principal component. Moreover, bootstrapped resampling was applied to AEQ scores and to Dysphoria and Affirmation levels from the UGDS-GS to refine data interpretation. Bootstrap is a statistical technique that allows building a representative distribution of a set of observed data by means of resampling with repetition (Hesterberg 2011, Wright *et al*. 2011). After selecting the number of resamples (*N* = 10 000), the bootstrapping procedure involved resampling the data *N* times and generating *N* simulated data points by averaging each resampled set. Applying the bootstrap approach to the AEQ scores, which are discrete by definition, yields a resampled distribution of continuous values, more suitable for the aims of our analysis. This approach can also be exploited for correlational or clustering measures (Wasserman *et al*. 1989). To assess significance when comparing bootstrapped distributions, it is necessary to examine the absence of overlap between their confidence intervals (CIs), rather than relying on parametric statistical tests.

## Results

Differences in the responses to the illusion statements between transgender participants who had received gender-affirming hormone therapy and those who had not were tested using the Mann–Whitney *U* test, and no significant differences emerged. This allowed us to determine that, in our sample, hormone therapy did not significantly affect embodiment magnitude. Virtual embodiment was assessed in all participants by comparing each illusion statement with its corresponding control. Statements targeting body ownership (Q1, Q2) and agency (Q5, Q6) yielded median scores above zero in both conditions, while the corresponding controls were close to or below zero. Consistent with previous embodiment research (Pyasik *et al*. 2023), this pattern indicates that participants effectively experienced the embodiment procedure (see Table 1 in Supplementary Materials). Median scores concerning ownership of the avatar’s mirror reflection and identification with its appearance were close to zero in the gender-congruent condition and below zero in the gender-incongruent condition, across both groups.

### Nonbootstrapped analyses

Illusion scores were further compared within groups and across conditions using Wilcoxon signed-rank tests, and between groups using the Mann–Whitney *U* test. We did not observe significant differences across any of the three leading AEQ dimensions (Ownership, Mirror Ownership, and Agency).

**Figure 2:**
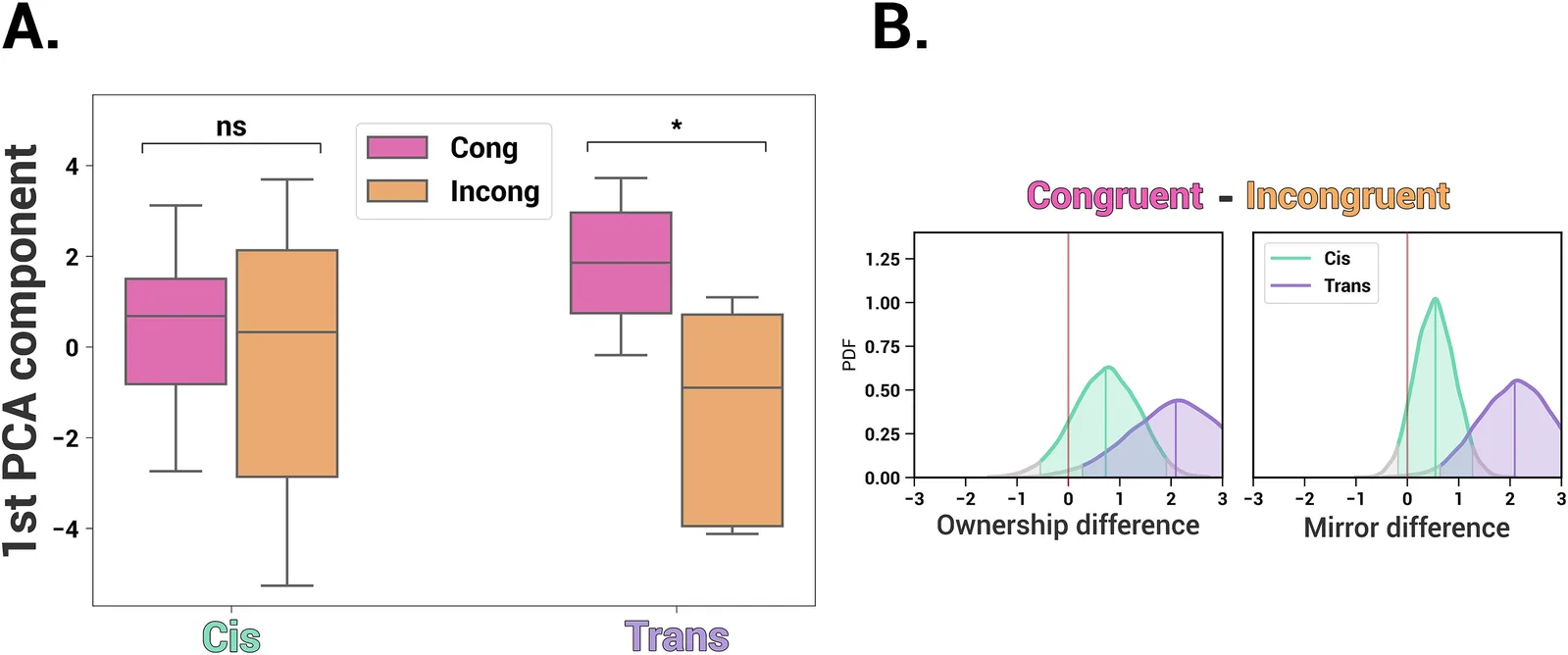
Congruent *versus* Incongruent embodiment differences. (A) Nonbootstrapped comparison of embodiment magnitude (represented by the first PCA component) between conditions in the two groups. In the Trans population, the magnitude of embodiment significantly differs (*P* < .05, FDR-corrected). (B) Bootstrapped differences between congruent and incongruent conditions for Cis and Trans groups along single embodiment dimensions. Above-zero differences can be observed in the Trans group along the Ownership (median: 2.09, CI: [0.18, 3.73]) and Mirror dimensions (median: 2.09, CI: [0.55, 3.46]).

PCA was then conducted following two separate pipelines. First, we defined distinct spaces for the *cis* and *trans* groups and compared the magnitude of embodiment between congruent and incongruent conditions within each group. Second, we defined separate spaces for each condition and compared the magnitude between groups. The first PCA component accounted for 61.8% of the variance in the *Cis* space and 62.3% in the *Trans* space. Within these spaces, differences in the magnitude of the embodiment between the congruent and incongruent conditions were assessed using a Wilcoxon signed-rank test, FDR-corrected. In the *Cis* space, the difference did not reach significance. In contrast, in the *Trans* space, we observed a significantly higher embodiment magnitude in the congruent condition than in the incongruent condition (*P* < .05, *W* = 8, Cohen’s *D* = 1.31, FDR-corrected) (see Fig. 2A). Across groups, embodiment magnitude did not differ significantly between participants in either condition.

### Correlations with UGDS-GS

According to a Mann–Whitney *U* test, UGDS-GS scores were significantly higher in the *Trans* than in the *Cis* group on both the Affirmation (*P* < .01, *W* = 17, Cohen’s *D* = −1.50, FDR-corrected) and Dysphoria (*P* < .001, *W* = 0, Cohen’s *D* = −3.77, FDR-corrected) subscales (Fig. 4A). To quantify the relationship between embodiment and the UGDS-GS subscales, Pearson correlations were calculated between each of the three embodiment measures and the Dysphoria and Affirmation scores for each subject, separately for each group and condition. No significant correlations were observed for either Dysphoria or Affirmation.

### Bootstrapped analyses

**Figure 3:**
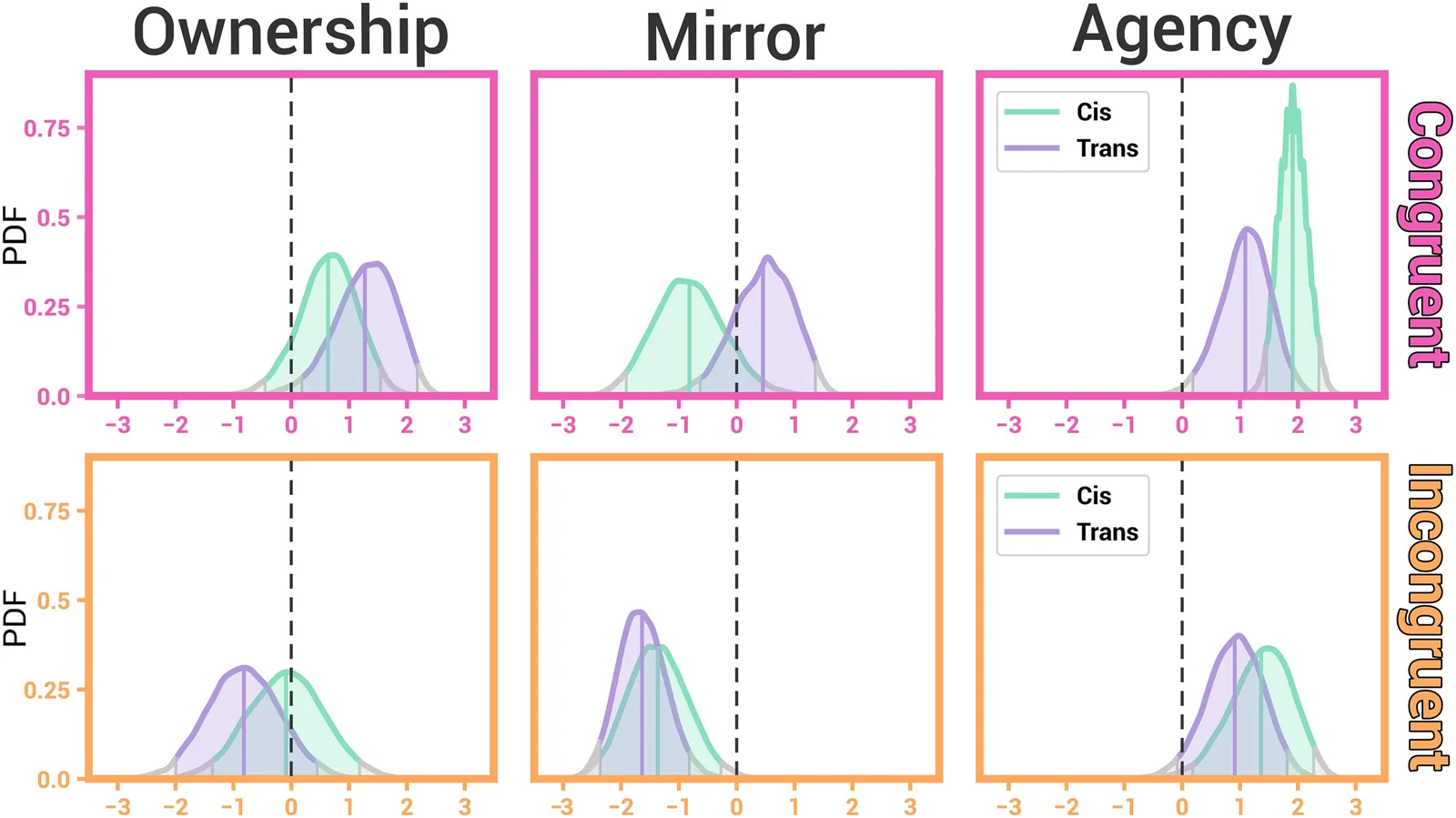
Bootstrapped AEQ dimensions. Bootstrapped evaluation of the three AEQ dimensions, namely, Ownership, Mirror Ownership, and Agency, for Cis and Trans groups in the two experimental conditions (Congruent and Incongruent avatars).

Bootstrap analyses (described in Materials & Methods) were performed with *N* = 10 000 resamples. In the congruent condition, bootstrapped single-dimension AEQ evaluation revealed significant above-zero values for Ownership (median: 1.27, CI: [0.18, 2.18]) and Agency (median: 1.09, CI: [0.18, 1.91]) in the *Trans* group, while only for Agency (median: 1.91, CI: [1.46, 2.36]) in the *Cis* group. With regard to the incongruent condition, we observed significant above-zero bootstrapped AEQ scores for Agency in the *Cis* group (median: 1.36, CI: [0.18, 2.27]) (Fig. 3).

Bootstrapped differences in embodiment magnitude were evaluated across groups and conditions for each of the three embodiment dimensions. In the *Trans* group, differences in a specific AEQ dimension experienced in congruent and incongruent conditions (i.e. congruent minus incongruent) have been observed for Ownership (median: 2.09, CI: [0.18, 3.73]) and Mirror Ownership (median: 2.09, CI: [0.55, 3.46]) dimensions, with higher embodiment levels in the Congruent condition (Fig. 2B). No significant differences were found between *Cis* and *Tran*s participants.

As for the nonbootstrapped data, we created four different 3D embodiment spaces, two divided by group and two by condition (Fig. 5A1 and B1). The *Trans* population shows a significant separation in the embodiment space between the congruent and incongruent condition, as shown by the nonoverlapping bootstrapped distribution of the first PCA component of illusion statement scores (Congruent median: 1.53, Congruent CI: [0.13, 2.58]; Incongruent median: −1.48, Incongruent CI: [−2.71, −0.27]) (Fig. 5B2) and from the silhouette score computed on the two clusters of points of the two conditions, which significantly differs from zero (*Trans*-space silhouette score median: 0.18, *Trans*-space silhouette score CI: [0.04, 0.43]) (Fig. 5C2). Conversely, we found no significant differences within the *Cis* group across conditions, both in the magnitude of embodiment (first PCA component; Fig. 5B2) and separation of the two distributions in the embodiment space (silhouette score; Fig. 5B3).

No significant between-subject differences emerged along the first PCA dimension, in the two spaces divided by experimental condition (Fig. 5A2). However, analysis of the silhouette score revealed a significant separation between *Cis* and *Trans* participants in the Congruent space (Congruent-space silhouette score median: 0.12, Congruent-space silhouette score CI: [0.00, 0.32]; Fig. 5A3). In the Incongruent space, the two clusters did not show a significant separation.

### Bootstrapped correlations with UGDS-GS

**Figure 4:**
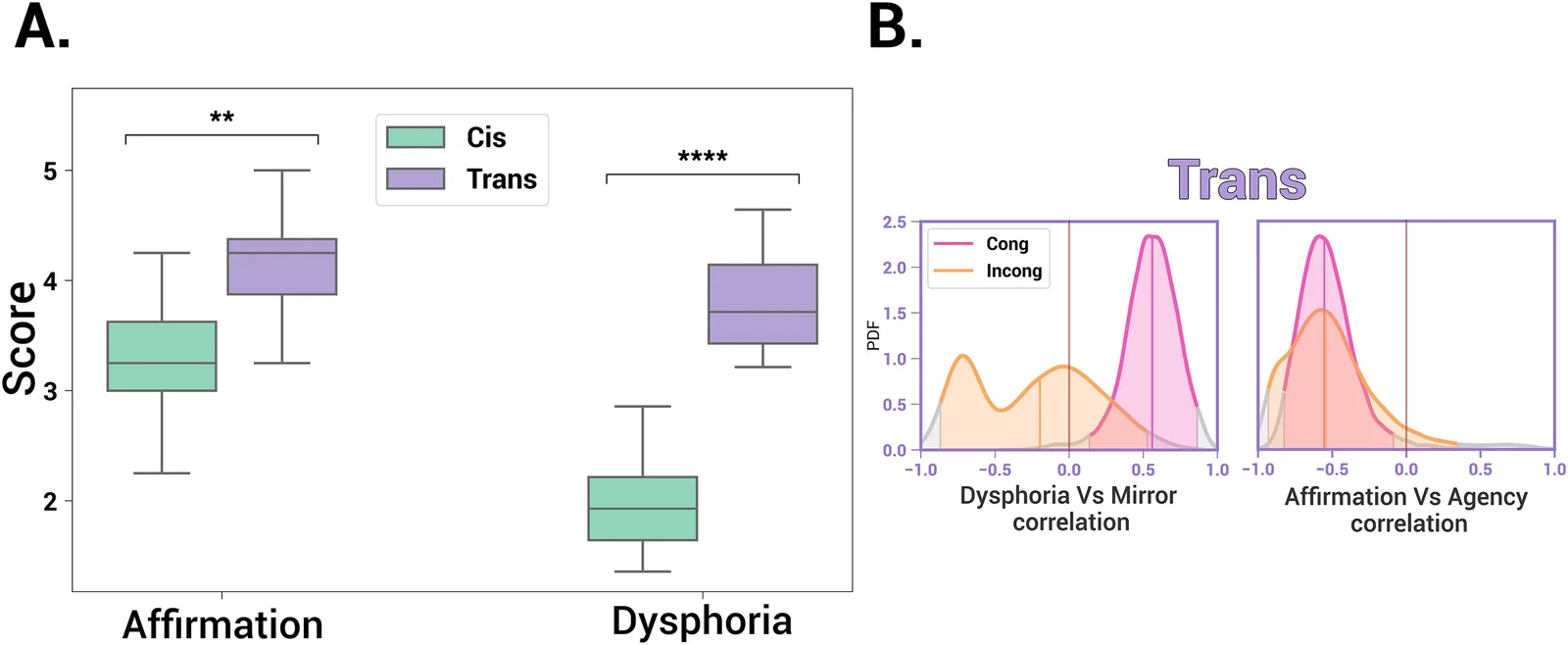
UGDS assessment and correlations with embodiment dimensions. (A) Nonbootstrapped comparison of Affirmation and Dysphoria levels between Cis and Trans groups. Both sets of scores significantly differ between groups (Affirmation, *P* < .01 FDR-corrected; Dysphoria, *P* < .001 FDR-corrected). (B) Bootstrapped correlation between UGDS scores and single embodiment dimensions. In the Trans group, a significant positive correlation was found between Dysphoria level and Mirror dimension (Pearson correlation median: 0.56, Pearson correlation CI: [0.16, 0.87]), while Affirmation levels negatively correlated with Agency dimension (Pearson correlation median: −0.55, Pearson correlation CI: [−0.83, −0.09]).

The bootstrapped correlation analysis between embodiment dimensions and Dysphoria showed a significant positive bootstrapped correlation regarding the Mirror Ownership dimension in the Congruent condition within the *Trans* group (Pearson correlation median: 0.56, Pearson correlation CI: [0.16, 0.87]; Fig. 4B). No other dimensions showed correlations with Dysphoria that reached significance.

Beyond Dysphoria, we also examined correlations with Affirmation. Notably, we found a significant negative correlation between Agency and Affirmation in the Congruent condition for the *Trans* group (Pearson correlation median: −0.55, Pearson correlation CI: [−0.83, −0.09]; Fig. 4B).

## Discussion

The present study investigated the effects of gender-aligned embodiment on perceived ownership—both from a 1PP and from an external, mirrored perspective—and agency in two groups of cisgender and transgender individuals during an FBI, to disentangle the reciprocal influence of gender identity and the bodily self.

Consistently with previous research on the FBI, we successfully induced the illusion of embodying a virtual avatar from a 1PP with visuomotor synchrony in all participants across both conditions. This effect was particularly robust in the *Cis* group, where no significant differences emerged between conditions in the single-dimension analyses, the PCA, or the bootstrap analyses. In contrast, within-subject results in the *Trans* group supported our initial hypothesis, showing that avatar gender alignment modulates the illusion’s strength.

**Figure 5:**
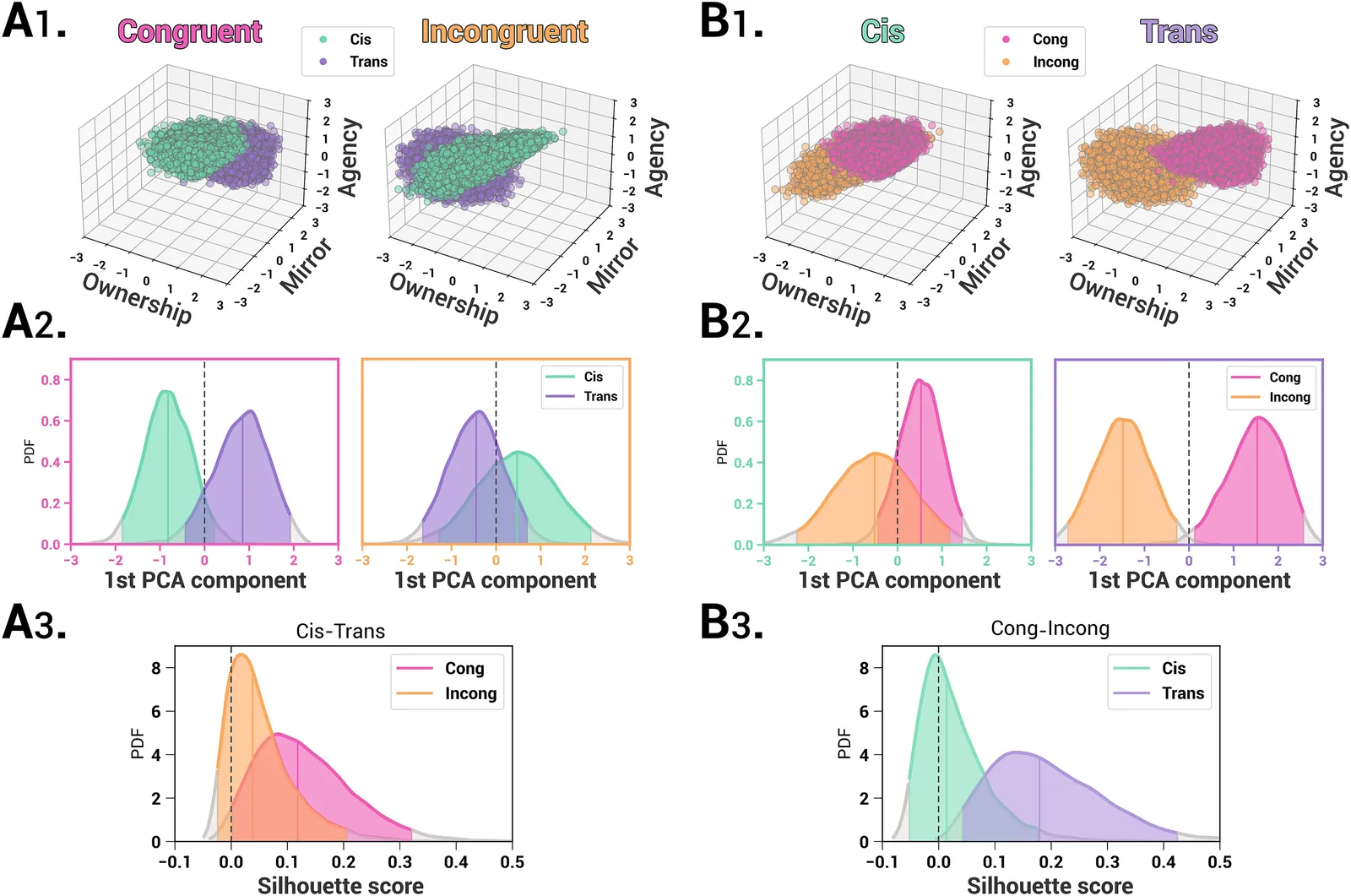
Bootstrapped embodiment spaces. (A1–B1) Three-dimensional representation of illusion statements, for each condition and group. (A2) Bootstrapped embodiment magnitude, represented by the first PCA component, in the condition-labeled spaces. (A3) Bootstrapped silhouette score of the condition-labeled spaces, showing between-subject differential values. Congruent differential scores were distributed significantly above zero (Congruent-space silhouette score median: 0.12, Congruent-space silhouette score CI: [0.00, 0.32]). (B2) Bootstrapped embodiment magnitude, represented by the first PCA component, in the group-labeled spaces. In the Trans group, we observed significantly separated distributions of the first principal component of the AEQ between conditions (Congruent median: 1.53, Congruent CI: [0.13, 2.58]; Incongruent median: −1.48, Incongruent CI: [−2.71, −0.27]). B3. Bootstrapped silhouette score in the condition-labeled spaces. In the Trans, we observed a significantly above-zero bootstrapped distribution of the silhouette score (Trans-space silhouette score median: 0.18, Trans-space silhouette score CI: [0.04, 0.43]).

Specifically, analyses of the first principal component—an informative and exhaustive dimension of embodiment magnitude—and of the distribution of bootstrapped AEQ scores across the single dimensions showed that congruency between the participant’s and the avatar’s gender significantly enhanced the illusion, regardless of sex assigned at birth. This pattern is particularly evident when considering: (i) the goodness of clustering of AEQ scores in the 3D embodiment space and its corresponding 1D projection onto the first principal component, as confirmed by the silhouette scores assessing within-group cluster quality across conditions (see Fig. 5B), and (ii) the distribution of bootstrapped congruent–incongruent differential scores in the two ownership dimensions (first-person ownership and mirror-ownership) (see Fig. 2B), which shows that embodiment strength across these two measures is higher in the identity-congruent condition for the *Trans* population. Beyond the specific effects observed in transgender participants, the present findings may have broader implications for understanding the relationship between gender identity and bodily self-consciousness. Because the Full-Body Illusion emerges from the integration of congruent multisensory signals, the selective enhancement of embodiment observed for gender-congruent avatars is compatible with the possibility that gender identity contributes to how bodily signals are integrated during embodiment, rather than merely influencing the subjective evaluation of bodily experience after multisensory integration has occurred. Under this interpretation, gender identity may represent one of several factors contributing to the subjective experience of embodiment, influencing the extent to which a virtual body is experienced as one’s own. This view is consistent with phenomenological accounts describing gender as a deeply embodied dimension of selfhood rather than a purely conceptual or narrative construct (Fausto-Sterling 2012). From this perspective, gender identity and multisensory integration may be understood as mutually constraining components of bodily self-consciousness. Therefore, the present findings are compatible with the hypothesis that aspects of gender identity may be instantiated, at least in part, within the same bodily mechanisms that contribute to the construction of the minimal self (Blanke and Metzinger 2009).

Within the Trans group, evidence for ownership emerged primarily in the congruent condition, particularly in the bootstrapped analyses. Agency, however, showed a different pattern. Agency scores were significantly above zero in both groups in the congruent condition and remained significantly above zero in the *Cis* group in the incongruent condition (Fig. 3), indicating that participants generally continued to experience control over the avatar’s movements. This dissociation suggests that ownership may be more strongly modulated by avatar gender congruency than agency (Fig. 3).

Exploratory correlational analyses in the TGD population suggest a potential relationship between individual differences in gender-related experiences and the embodiment illusion: as dysphoria increases, the perceived ownership of the congruent virtual body seen in the mirror also increases; as affirmation increases, the sense of agency experienced with the congruent avatar decreases. The significant correlation between the mirror dimension of the AEQ and the Dysphoria subscale of the UGDS-GS is particularly noteworthy, as mirror-related embodiment requires a shift in perspective from first to third person. This exploratory finding suggests that individual differences in gender dysphoria may be associated with how transgender participants experience and evaluate their embodied appearance when viewed through the virtual mirror. Conversely, higher levels of affirmation were associated with lower agency scores in the congruent condition, indicating that developing a more harmonious experience of selfhood through gender affirmation may relate differently to the subjective components of embodiment. However, the mechanisms underlying these associations remain speculative and require further investigation.

These correlations shed new light on the relationship between sensing ownership of the mirror-reflected body that is attributed to the self, and the sense of agency. In particular, the process described here seems to go beyond the state-of-the-art description of embodiment. Previous studies successfully dissociated ownership and agency over a synthetic hand, where different forms of “incongruency,” like implausibility of the rubber hand’s position (Kalckert and Ehrsson 2012, Marotta *et al*. 2017), or it being located outside of the hand-reachable space (Mangalam *et al*. 2019), abolish ownership but not agency. Instead, our findings suggest that gender congruency may influence both dimensions of embodiment—the sense of ownership toward the mirror-reflected body and the sense of agency over the avatar’s actions—during a virtual embodiment illusion. Nevertheless, the present design does not allow direct conclusions regarding the hierarchical relationship between ownership and agency; for this reason, results should be interpreted with caution. Overall, cisgender and transgender individuals experienced the FBI similarly. However, the two groups show different extents of embodiment of the congruent avatars, as shown by the geometric distance of embodiment in the 3D AEQ space, where the clusters associated with the two groups in the congruent condition show a silhouette score significantly different from zero (Fig. 5A3). This result suggests that, in the congruent condition, the illusion of embodying the avatar was stronger in transgender than in cisgender participants. However, this between-group difference reflects the within-group effect observed in the *Trans* group, suggesting that both transgender and cisgender individuals embody gender-congruent and gender-incongruent avatars in comparable ways. Still, gender congruency selectively amplifies the illusion in transgender participants.

Previous theoretical accounts have suggested that alterations in bodily self-experience may emerge from changes in the integration of multisensory bodily signals (Ciaunica *et al*. 2022). Within this framework, the present findings raise the possibility that gender identity may act as an important constraint on the multisensory processes that support bodily self-consciousness.

### Limitations & future directions

While our results provide valuable insights into the mechanisms of virtual embodiment, certain methodological and conceptual limitations must be considered. The present study is conceptually oriented toward a transfeminist-informed approach to neuroscience, aiming to create depathologizing and trans-positive knowledge in the field, taking inspiration from the works of the Neurogenderings Network (DuBois *et al*. 2025). Nevertheless, it is necessary to point out that the experimental design is flawed in many aspects due to its binary structure. This represents an important limitation of the present study, as the binary categorization of both participants and avatars does not fully reflect the theoretical framework of gender diversity and gender/sex entanglement outlined in the Introduction. The general approach has been that of contrasting dichotomies: sex ‘versus’ gender-related factors, cisgender versus transgender, and typical versus atypical. Since “sex and gender are neither dichotomous nor independent from each other” (Fausto-Sterling 2019), future research could shift from an interactionist perspective—i.e. tracing a strong separation between sex-related and gender-related factors that interact with each other to produce various developmental outcomes—to an ‘entanglement’ perspective, for which it is virtually impossible to identify a factor as strictly sex- *or* gender-related but rather “co-constituted and in dynamic, looping relationships, with every factor understood as being both sex-and-gender at the same time” (Ritz *et al*. in DuBois *et al*. 2025, p. 98). For the same reasons, future research could avoid binary group comparisons and instead favor spontaneous cluster emergence from data (Ritz *et al*. in DuBois *et al*. 2025, p. 96; Bryant *et al*. 2019).

The FBI is indeed a powerful tool for manipulating bodily representations, but when self-identification is taken into consideration, stereotypical and polarized virtual avatars may be limited in tackling identity alignment. Indeed, an important limitation of this study has been the use of preconfigured avatars with highly dimorphic features, restricting its capacity to capture the participant’s self-identification with their own affirmed gender. Future research could overcome this limit by using self-tailored avatars.

Furthermore, the present study rejected bioessentialist sample sorting methods (i.e. categorization based on sex assigned at birth) in favor of what DuBois *et al*. (2025) define as “individualized gender fixation,” namely, operationalizing only self-reported gender identity. This approach could limit scientific rigor and standardization, undermining future replicability. Future research should introduce novel approaches to operationalize gender identity that, for example, could capitalize on the potential of virtual settings to quantify multidimensional, out-of-the-binary identity alignment.

On a final note, this work focused on phenomenological, subjective, and explicit reports of the virtual embodiment experience. Accordingly, all primary outcome measures relied on self-report questionnaires, which may be influenced by response biases, demand characteristics, and individual differences in introspective abilities. Despite its inherent richness in reflecting how the subject lives the illusion, methods like the AEQ are limited in assessing the subject’s implicit sense of ownership and agency. Future studies could introduce implicit methods to assess both dimensions in a virtual environment, to fully capture their intrinsic prenoetic nature.

## Conclusions

In this paper, we examined how both cisgender and transgender individuals sense ownership, mirror-ownership, and agency over a virtual avatar, to shed further light on the relationship between bodily self-consciousness and gender identity. The phenomenological evidence on the subjective experience of embodying a gender-congruent versus gender-incongruent avatar indicates that affirmed gender identity in TGD individuals, rather than own-body perception or sex assigned at birth, is associated with a stronger magnitude of embodiment when the FBI involves a gender-congruent avatar. Alongside this affirmation-oriented pattern, exploratory analyses suggested a possible association between embodiment strength and the level of gender dysphoria, a finding that should be interpreted with caution. Our findings challenge the initial hypothesis regarding a potential tendency toward disembodiment in TGD individuals. In contrast, the embodiment procedure was successful across all conditions, and ownership and agency scores did not differ significantly from those of cisgender participants. This suggests that transgender embodiment may, in fact, be grounded, and possibly reinforced, by the awareness of alignment with one’s affirmed gender. Overall, our findings suggest that gender identity may be closely related to the multisensory processes underlying bodily self-awareness, leading us to rethink how beliefs about the gendered body are formed and sustained.

## Supplementary data

Supplementary data are available at *Neuroscience of Consciousness* online.

## Supplementary Material

Supplementary_niag047

## Data Availability

Data are available upon request to the corresponding author.
