# Correction: Affirmed gender shapes virtual embodiment: a phenomenological account of ownership and agency across gender identities

**DOI:** 10.1093/nc/niag057

**Published:** 2026-08-24

This is a correction to: Marina Leggieri, Alessio Borriero, Tommaso Ciorli, Maria Pyasik, Selene Schintu, Sarah Finzi, Maria Teresa Molo, Lorenzo Pia, Affirmed gender shapes virtual embodiment: a phenomenological account of ownership and agency across gender identities, *Neuroscience of Consciousness*, Volume 2026, Issue 1, 2026, niag047, https://doi.org/10.1093/nc/niag047

In the originally published version of the paper, the first and last names of all except the first co-authors were erroneously transposed. This error is now corrected in the article.

