# Supplementary material for: Affirmed gender shapes virtual embodiment: a phenomenological account of ownership and agency across gender identities
Source: Neurosci Conscious. 2026 Aug 14;2026(1):niag047. doi: 10.1093/nc/niag047 (PMC13474268; doi:10.1093/nc/niag047)
Supplement: Supplementary file 1 [file supplementary_niag047.docx]

## Supplementary Materials

For

Affirmed Gender Shapes Virtual Embodiment: A Phenomenological Account of Ownership and Agency Across Gender Identities

**Table 1| Avatar Embodiment Questionnaire statements and descriptives across groups and conditions.**
